# The Halogen Bond in Weakly Bonded Complexes and the Consequences for Aromaticity and Spin-Orbit Coupling

**DOI:** 10.3390/molecules28020772

**Published:** 2023-01-12

**Authors:** Ana V. Cunha, Remco W. A. Havenith, Jari van Gog, Freija De Vleeschouwer, Frank De Proft, Wouter Herrebout

**Affiliations:** 1 MolSpec, Departement Chemie, Universiteit Antwerpen, Groenenborgerlaan 171, 2020 Antwerpen, Belgium; 2 Stratingh Institute for Chemistry and Zernike Institute for Advanced Materials, Rijksuniversiteit Groningen, Nijenborgh 4, 9747 AG Groningen, The Netherlands; 3 Gent Quantum Chemistry Group, Faculteit Wetenschappen, Universiteit Gent, Krijgslaan 281 (S3), 9000 Gent, Belgium; 4 Algemene Chemie (ALGC), Vrije Universiteit Brussel (VUB), Pleinlaan 2, 1050 Brussel, Belgium

**Keywords:** halogen bonds, density functional theory, energy decomposition analysis, ring current analysis, spin-orbit coupling

## Abstract

The halogen bond complexes CF${}_{3}$X⋯Y and C${}_{2}$F${}_{3}$X⋯Y, with Y = furan, thiophene, selenophene and X = Cl, Br, I, have been studied by using DFT and CCSD(T) in order to understand which factors govern the interaction between the halogen atom X and the aromatic ring. We found that PBE0-dDsC/QZ4P gives an adequate description of the interaction energies in these complexes, compared to CCSD(T) and experimental results. The interaction between the halogen atom X and the π-bonds in perpendicular orientation is stronger than the interaction with the in-plane lone pairs of the heteroatom of the aromatic cycle. The strength of the interaction follows the trend Cl < Br < I; the chalcogenide in the aromatic ring nor the hybridization of the C–X bond play a decisive role. The energy decomposition analysis shows that the interaction energy is dominated by all three contributions, viz., the electrostatic, orbital, and dispersion interactions: not one factor dominates the interaction energy. The aromaticity of the ring is undisturbed upon halogen bond formation: the π-ring current remains equally strong and diatropic in the complex as it is for the free aromatic ring. However, the spin-orbit coupling between the singlet and triplet $\pi \to \pi^{*}$ states is increased upon halogen bond formation and a faster intersystem crossing between these states is therefore expected.

## 1. Introduction

Van der Waals interactions are omnipresent in nature, and are crucial for understanding the dynamics and structure of a wide variety of systems. A particularly weak interaction is the halogen bond. The International Union of Pure and Applied Chemistry (IUPAC) defines a halogen bond as “a net attractive interaction between an electrophilic region associated with a halogen atom in a molecular entity, and a nucleophilic region in another, or the same, molecular entity” [1]. In 1961, Zingaro et al. [2] described complexes formed in solution by halogens and phosphine oxides and sulfides. This was the first time that the term “halogen bond” was used to describe interactions where halogens act as electrophilic species. However, it was much later that Glaser et al. [3] suggested to use the term halogen bond to describe an interaction between halogen atoms, regardless of their electrophilic or nucleophilic nature. It was in 2009 that the IUPAC gave a unified conceptual framework for the interactions involving halogens [1]. This interaction, which captured the attention of the scientific community for decades, can be discussed in terms of its unique features, such as directionality, tunability, hydrophobicity, and the atomic radius of the donor atom. Halogen bonds (XB) of the form R–X⋯Y, with X the halogen bond acceptor and Y the donor, are particularly directional interactions, due to the localization of the σ-hole exactly on the elongation of the covalent bond that the halogen atom is involved [4]. The effective atomic size along the extended R–X bond axis, in monovalent halogen atoms, is smaller than in the direction perpendicular to this axis [5,6,7]. This corresponds to a region of depleted electron density, the so-called σ-hole. Thus, shorter and stronger halogen bonds are more directional than longer and weaker counterparts. However, the directionality of the XB acceptor is along the axis of the donated lone pair on Y [8,9,10,11,12,13,14,15,16,17,18,19]. For the case in which the XB acceptor is an isolated π-system, the axis of the R–X bond is approximately along the axis perpendicular to the π-system. Furthermore, when the XB acceptor contains both lone pairs and aromatic π-pairs, the lone pairs are generally the ones involved in the XB formation. However, the two exceptions are furan and thiophene, for which both the lone pairs and the π-bonding orbitals are involved in the XB formation; the hydrogen bond interactions with the lone pairs or the π-bonds are of similar strength [20]. Tunability is another feature of this interaction, in which the XB ability increases on the order I > Br > Cl > F. This trend is often concordant with the positive character of the σ-holes [21], which decreases with the electronegativity of the halogen atom, but increases with the polarizability [22,23,24,25]. Thus, the donor ability of a given compound can be tuned by selecting a halogen atom as a donor site [26,27]. However, the tuning of the σ-hole magnitude can also be achieved by modifying the hybridization of the carbon bond to the XB donor site. Thus, the strength increases in the following order: C(sp)-X > C(sp${}^{2}$)-X > C(sp${}^{3}$)-X, and it has been observed for various systems [16,19,28,29,30,31,32]. Hence, there are three main possibilities to tune the XB interaction strength: (I) by single atom mutation; (II) by changing the hybridization; and (III) by modifying the electron withdrawing groups [33,34,35,36,37]. The size of the donor atoms influences the steric hindrance, as halogen atoms have large van der Waals radii. Thus, when compared to the hydrogen bond, the XB bond is more sensitive to steric effects [38,39,40]. This has been shown in the formation of DNA pairs where HB is replaced by XB. The latter shows that bromine gives more stable pairs when compared to iodine, due to the steric repulsions arising from the larger radius of the I atom [41,42,43,44,45,46,47,48,49,50]. Furthermore, these halogen bonds also impact optical transitions of supramolecular complexes, that can undergo singlet to triplet intersystem crossings [25,51,52,53,54,55,56,57]. The size of halogen atoms plays an important role in the photoluminescence of halogenated chromophores. This is shown in reference [58], in which the singlet to triplet intersystem crossing rate increases by a factor of 60 when using iodine corroles instead of fluorine ones. Hence, the heavy atom effect confers to XB-based materials particularly exciting promising applications. Such applications have been demonstrated in [52,53,54,55,59,60,61], in which halogen bonding was used to tune room-temperature phosphorescence in organic crystals. A large number of studies showed the use of halogen bonds for changing the electronic properties in numerous types of materials, ranging from crystalline solids to amorphous [56,57,60,62,63,64,65,66]. Furthermore, XB bonds have also proven to be suitable for changing the efficiency of solar cells, and perovskites. However, the modelling of these systems is still a bottleneck for computational chemistry.

Most of the electronic structure methods used for molecular modelling capture 99% of the total electronic energy [67]. However, the remaining missing fraction, which is crucial for molecular properties, such as relative energies [68,69,70,71] and binding properties [72,73,74,75], arise from correlated motion of electrons [76,77,78]. The main component of this energy is the long-range contribution, also known as van der Waals (vdW) or dispersion interactions [70,79]. These forces are dominant in weakly bonded complexes, and, thus, the modeling of these systems requires the introduction of dispersion corrections [80,81,82,83], or correlated wavefunction methods.

**Figure 1:**
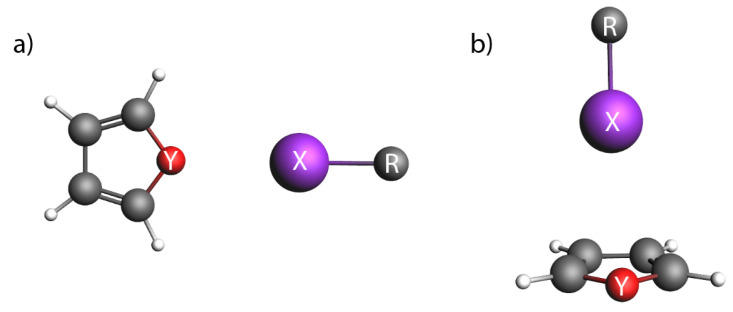
Schematic Yes you can move it. representation of geometries in (**a**) parallel (‖), and (**b**) perpendicular (⊥) orientation. X = Cl, Br, I; Y = O, S, Se; R = CF${}_{3}$, C${}_{2}$F${}_{3}$.

In this study, we present a detailed bonding analysis of halogen bonding in complexes consisting of a small molecule (CF${}_{3}$X/C${}_{2}$F${}_{3}$X (X = Cl, Br, I)) and an aromatic molecule (furan/thiophene/selenophene) in various orientations. These complexes provide insights into the structure of halogenated crystals. The orientations that we consider are the halogen bonds with the lone pair (parallel orientation) and with the π-bonds (perpendicular orientation) of the chalcogenide of the aromatic ring (Figure 1). We also study the effect of a halogen bond on the electronic transitions of the aromatic molecule and possible intersystem crossing due to increased spin-orbit coupling as a consequence of the vicinity of a heavy atom. We use DFT and CCSD(T) calculations to retrieve the effects of correlation, and decompose the interaction energies into their different contributions. In this way, we are able to investigate several factors affecting the halogen bond, namely, the halogen atom of the donor, the aromatic ring as the acceptor, the hybridization of the carbon atom to which the halogen atom is attached, and the orientation of the halogen bond (parallel or perpendicular). Moreover, we are able to benchmark dispersion-corrected DFT and judge its suitability to describe these weakly interacting systems with halogen bonds. Finally, we investigate whether the formation of the halogen bond affects the aromaticity of the ring, and if it affects the $\pi \to \pi^{*}$ transition in the aromatic ring, and the spin-orbit coupling between the singlet and triplet $\pi \to \pi^{*}$ states.

## 2. Results and Discussion

### 2.1. Interaction Energies at Different Levels of Theory

**Figure 2:**
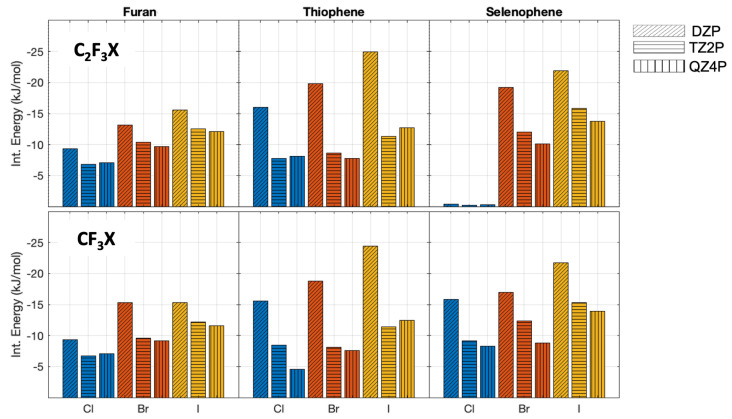
PBE0−dDsC We changed it interaction energies (without zero-point vibrational energy correction) with different basis sets for the complexes oriented in parallel. The different halogens are depicted with the following colormap: chlorine blue, bromine red, and iodine yellow. The DZP basis set is represented with slanted lines, the TZ2P with horizontal lines, and QZ4P with vertical lines.

The interaction energies obtained with different basis sets, for the complexes in the parallel orientation, are shown in Figure 2. The interaction energies (without zero-point vibrational energy correction) change significantly with the increase of the basis set size, with differences ranging between 0.1 to 13.6 kJ/mol (Figure 2). It shows, as expected, the importance of the use of a large basis set for weakly bonded complexes. Note that for C${}_{2}$F${}_{3}$Cl⋯selenophene in parallel orientation, hardly any interaction is found with all three basis sets. However, the differences in the interaction energies obtained by using the TZ2P and QZ4P basis sets are considerably smaller and range only from 0 to 3.9 kJ/mol; this indicates that the interaction energy is close to convergence with a TZ2P basis set, showing that the QZ4P basis therefore is certainly sufficiently large enough.

**Figure 3:**
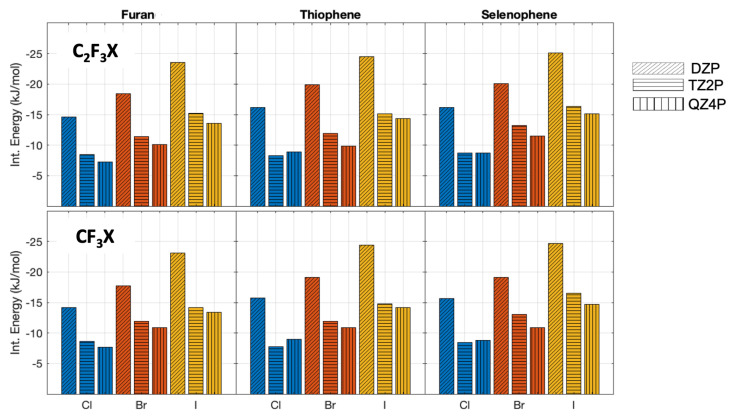
PBE0−dDsC interaction energies (without zero-point vibrational energy correction) with different basis sets for the complexes oriented in perpendicular. The halogen atoms are depicted with the following colormap: chlorine blue, bromine red, and iodine yellow. The DZP basis set is represented with slanted lines, the TZ2P with horizontal lines, and QZ4P with vertical lines.

For the perpendicular oriented complexes, the same trend is observed (Figure 3). The interaction energies calculated with the DZP basis set differ at most 10.2 kJ/mol and at least 6.5 kJ/mol. The differences between the interaction energies evaluated by using the TZ2P and QZ4P basis sets decrease again. Here, the largest difference is 2.1 kJ/mol and the minimum difference calculated is 0 kJ/mol (supplementary information). Taking into account the small differences between the interaction energies with a TZ2P and QZ4P basis set, we can conclude that the QZ4P basis set is of sufficient quality. Hence, this basis set will be used for the EDA calculations.

Although the DFT results are robust with basis set size, an important question remains to be answered: “Does the PBE0 functional with dispersion corrections give a reasonable description of these weak interacting complexes?” Thus, we have calculated the interaction energies also by using the CCSD(T)-DLPNO/CBS approach. For both the parallel oriented complexes (Table 1) and the perpendicular oriented complexes (Table 2), the differences between the CCSD(T) interaction energies and the DFT ones are largest for the iodine halogen bonds but do not vary more than 5 kJ/mol. Hence, PBE0-dDsC/QZ4P is suitable to describe the weak bonding interactions in these complexes and captures most of the relevant physics.

A last benchmark of the theoretical data is a comparison of the calculated interaction enthalpies with experimentally determined enthalpies (Table 3) for CF${}_{3}$I⋯furan. For the compounds for which experimental data are available, the agreement between theory and experiment is good, and only small deviations are found, which are within chemical accuracy. Thus, the PBE0-dDsC/QZ4P calculations are of sufficient quality and in the following sections only the results obtained with PBE0-dDsC/QZ4P are discussed and the remaining results can be found in the supplementary information.

### 2.2. Interaction Energies and Trends

The hard–soft acid–base (HSAB) principle states that soft acids preferably interact with soft bases, while hard acids prefer interacting with hard bases, when all other factors are equal. Thus, when oxygen is switched to selenium in a molecule, the size of the atom increases as well the polarizability; however, the electronegativity decreases. Therefore, the oxygen can be considered as a hard base and selenium as a soft base, and the sulfur is in between. The same reasoning can be made for the halogen atoms, which results in the following ranking of their softness as acids: Cl < Br < I. The delocalized π-systems in the heteroaromatic molecules act as Lewis bases and are considered to be soft while the lone pairs present on the heteroatoms are harder. Based on the HSAB principle, it is expected that complexes having the lowest interaction energy contain the following combination of halogen and chalcogen atoms: chlorine (hard) and selenium (soft) or iodine (soft) and oxygen (hard). The strongest interaction, on the other hand, is expected to occur between complexes containing chlorine and oxygen or iodine and selenium. For the complexes corresponding to a perpendicular geometry, where the halogen atom interacts with the soft, delocalized π-system, the strongest interacting complex is expected to contain a soft iodine atom. The complex with the lowest interaction energy in perpendicular position, however, can be predicted to accommodate a hard chlorine atom. Based on the σ-hole theory, which considers only electrostatic interactions, a difference is expected between the sp${}^{2}$ and sp${}^{3}$ hybridized complexes: the carbon atom with sp${}^{2}$ hybridization is expected to be more electronegative (higher s-character), and thus these complexes are expected to have a larger electrostatic interaction for the same halogen atom than their sp${}^{3}$ hybridized counterparts. Furthermore, as the halogen atom is changed from chlorine to bromine, and further to iodine, the polarizability of the halogen atom increases, and its electronegativity will decrease. The increase in polarizability is expected to lead to an increased σ-hole and subsequently to a larger electrostatic contribution. Moreover, going down in the periodic table will also lead to an increased dispersion interaction. In addition to these longer-range interactions, Pauli repulsion and potentially charge transfer may also play their part, as demonstrated in a study of various CX${}_{3}$I and halide anion interactions [21]. Hence, which effect will dominate is not clear based on qualitative arguments, as has been found previously [84].

These hypotheses can be tested by carrying out an EDA analysis on the PBE0-dDsC/QZ4P interaction energies ($E_{\mathrm{bond}}$) for all complexes, presented in Table 4 and Table 5. The total bonding (interaction) energies are decomposed in contributions of the Pauli repulsion term ($E_{\mathrm{Pauli}}$), electrostatic interaction ($E_{\mathrm{els}}$), the steric interaction ($E_{\mathrm{steric}}=E_{\mathrm{Pauli}}+E_{\mathrm{els}}$), the orbital interaction term ($E_{\mathrm{orb}}$), and the dispersion ($E_{\mathrm{disp}}$) term.

A first glance at the interaction energies in parallel orientation (Table 4) shows that indeed the weakest interacting complex is C${}_{2}$F${}_{3}$Cl⋯selenophene, as predicted by applying the HSAB argumentation. The strongest interacting species are C${}_{2}$F${}_{3}$I⋯selenophene and CF${}_{3}$I⋯selenophene, also inline with the HSAB rules. However, the interaction in CF${}_{3}$Cl⋯selenophene, predicted to be weak, are on par with other species. Moreover, the interaction in C${}_{2}$F${}_{3}$Cl⋯furan, predicted to be strong, is only half as strong as the interaction in C${}_{2}$F${}_{3}$I⋯furan. For the perpendicular cases (Table 5), the I⋯Se interactions are indeed the strongest, but the Cl⋯Se interactions, predicted to be weak, are stronger than the Cl⋯O interactions, predicted to be strong. This indicates that the HSAB principle does not tell the full story, and predictions based solely on these considerations can be substantially in error. In addition, the prediction based on the σ-hole theory—that the sp${}^{2}$ hybridized species interact more strongly than their sp${}^{3}$ counterparts—is not immediately confirmed by the DFT interaction energies. The differences between the sp${}^{2}$ and sp${}^{3}$ hybridized species is in general very small, with only a few exceptions in one case confirming and in one case disproving the σ-hole predictions. It is evident that predicting the interaction strength cannot be based purely on qualitative arguments and that the interaction is a more subtle interplay of different contributions.

One trend that is generally followed for all species is that when X is varied from Cl to I, the interaction energy increases, as is predicted from considering the larger polarizability of X when going down in the periodic table. This effect is much less pronounced when Y of the aromatic ring is varied from O to Se: much smaller variations in the interaction energies are discernible and an increasing trend is not always found. This highlights again that these interactions are not solely determined by one factor.

If we look at the EDA analysis in more detail, we notice that the total steric interaction, which is a sum of the Pauli repulsion and the electrostatic interaction, is for most cases (parallel and perpendicular) rather small and in many cases repulsive. The electrostatic interactions do not dominate the final interaction energy. The orbital interaction energy and the dispersion energy also contribute significantly to the interaction between the two molecules in the complex, and both are equally important. The dispersion energy follows the expected trend of increasing when going down in the periodic table, if the halogen atom is changed. This trend is not clearly visible if the heteroatom in the aromatic ring is changed from O to Se. The differences in the dispersion contribution for sp${}^{2}$ and sp${}^{3}$ species are small, and more notable when the parallel geometries are compared to the perpendicular ones. For the perpendicular cases, where the halogen atom interacts with the π-system, the dispersion interactions are in general larger.

The orbital interaction term is in nearly all cases substantial. The orbital interaction term has its origin here in the relaxation of the orbitals of both fragments due to mutual polarization and to a lesser extent charge transfer. Thus, for the heavier halogen atoms, these orbital interactions are stronger, as they are more polarizable. Trends in the variations in the orbital contribution term due to the change from sp${}^{2}$ to sp${}^{3}$, due to the change from O to Se, or due to the change from parallel to perpendicular are harder to detect, and an a priori rule for predicting the magnitude of this term based on qualitative considerations proves to be unreliable.

We found that in general the interactions between the molecules in perpendicular orientation are larger than in parallel orientation, but not one factor is dominating in this increase. In some cases, the dispersion contribution is larger, in other cases, the orbital interaction term. The nature of the aromatic ring (Y = O, S, or Se) plays a much less decisive role, and no clear trends are visible. The hybridization of the carbon atom to which the halogen atom is attached also does not govern the interaction energy. The only clear trend is with halogen atom, when it becomes larger (X = Cl, Br, I), the interaction energy becomes stronger, mainly due to polarization of the fragments.

**Figure 4:**
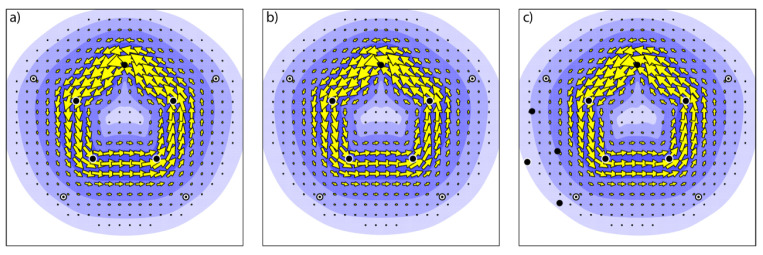
Plots of the π-current density for (**a**) furan, (**b**) CF${}_{3}$I⋯furan (‖), and (**c**) CF${}_{3}$I⋯furan (⊥).

As we have seen that the interactions between the halogen bond donor and the aromatic π-system can be relatively strong, and caused by mutual polarization (as shown by the orbital interaction term), two remaining issues have to be resolved. One is whether the changes, induced by halogen bond formation, in the orbitals are such that the aromaticity of the five-membered ring is influenced, and the second one is whether the presence of the heavy halogen atom and the accompanying changes in orbitals affect the optical properties of the five-membered ring, and the spin-orbit coupling between the lowest electronic states. To answer the first question, the π-current density, induced by an external magnetic field has been calculated. Plots of the induced π-current density for CF${}_{3}$I⋯furan in parallel and perpendicular geometry are presented in Figure 4, together with a plot of the induced current density for the free aromatic ring. The induced current density is plotted in a plane, 1 $a_{0}$ above the molecular plane of the five-membered ring. The plots are visually indistinguishable from each other, indicating that no major changes in aromaticity occur upon complexation. This is further corroborated by the $j_{max}$ values, the maximum strength of the current density. For furan, a $j_{max}$ value of 0.0855 a.u. is found, whereas for CF${}_{3}$I⋯furan (‖) a value of 0.0824 a.u. is found and for CF${}_{3}$I⋯furan (⊥) 0.0827 a.u. Hence, upon formation of the halogen bonds, the induced π-current density is unaffected, and the aromatic character of the ring remains unchanged. This result is expected based on the symmetry selection rules in the ipsocentric formulation [85,86], as the nodal structure of the π-orbitals is not influenced by the halogen bond formation.

For the exploration of the influence of the halogen bond on the excited states of the aromatic ring, we have selected several complexes (Table 6) with varying halogen (Br/I), orientation, and hybridization. The spin-orbit coupled excitation energies of the lowest, bright, $\pi \to \pi^{*}$ transition is barely influenced by the presence of the halogen bond. That also holds for the energy of the lowest triplet state that has $\pi \to \pi^{*}$ character. In all complexes, the three levels of the triplet remain degenerate, but in the perpendicular orientation, for the I⋯furan complexes, a very small splitting in the three levels is visible, due to the close proximity of the iodine atom to the π-system.

The spin-orbit coupling between the bright $\pi \to \pi^{*}$ state and the lowest triplet state is small for all aromatic rings. A negligible increase is discernible if the halogen atom forms a halogen bond in parallel orientation, but in the perpendicular orientation, the spin-orbit coupling is significantly increased. The increase in spin-orbit coupling follows the trend selenophene > thiophene > furan, but, unexpectedly, the spin-orbit coupling in C${}_{2}$F${}_{3}$I⋯furan is smaller than for the analogous Br complex, which may be caused by the closer proximity of the heavy atom to the π-system in the Br case than in the I case. Furthermore, despite the fact that in the I case a stronger mixing of the occupied π-system of the furan with the π-system of C${}_{2}$F${}_{3}$I occurs, a larger mixing in the unoccupied π-orbitals with σ-like C${}_{2}$F${}_{3}$Br orbitals occurs, which may enhance the spin-orbit coupling more in the Br case. An unexpectedly large spin-orbit interaction is observed for C${}_{2}$F${}_{3}$I⋯thiophene complex, with concomitantly a strong mixing of the C${}_{2}$F${}_{3}$I orbitals with the occupied and unoccupied π-orbitals of thiophene, which further enhances the spin-orbit interaction.

In all perpendicular cases, an increase in spin-orbit coupling matrix element is observed due to formation of the halogen bond, hence, intersystem crossings from singlet to triplet can be accelerated by the formation of halogen bonds with heavy atoms. Thus, triplet formation in π-conjugated molecules can be accelerated by adding iodine-containing additives that form halogen bonds with the π-systems. This effect may find an application in organic electronic devices when it is desirable to increase the formation of triplets.

## 3. Materials and Methods

The starting geometries of the weakly bonded complexes were built by using the Amsterdam Modelling Suite–Graphical User Interface (AMS-GUI). The aromatic molecule (furan/thiophene/selenophene) was complexed with a small molecule with sp${}^{2}$, and sp${}^{3}$ hybridization (C${}_{2}$F${}_{3}$X or CF${}_{3}$X, with X = Cl, Br, I), which were placed in a parallel and perpendicular orientation, Figure 1. This procedure rendered 36 different geometries.

Geometry optimizations were performed with the PBE0 functional (following the work of [87]), together with density-dependent dispersion corrections (dDsC) [88], and using the DZP, TZ2P, and QZ4P basis sets. Scalar relativistic effects were taken into consideration by using the ZORA formalism [89,90,91]. Frequency calculations were performed at the same level of theory for all basis sets to confirm that all stationary points are minima on the PES (see SI for a list of all frequencies for all molecules obtained with the QZ4P basis set). At the optimized geometries, the interaction energies were further analysed by using the energy decomposition analysis (EDA) [92]. These calculations were performed with the AMS-2022 suite [82,93,94]. Single-point energy calculations were also performed with the CCSD(T) DLPNO method [95], with the ORCA-5.070 package [96,97], with extrapolation to the complete basis set limit by using the def2-TZVP and def2-QZVP basis sets on the PBE0-dDsC/QZ4P optimized geometries. The aromaticity of the weakly bonded complexes was studied by using the magnetic criterion, and the magnetically induced current density was calculated by using Gamess-UK [98,99] and SYSMO [100] by using the PBE0 functional, def2-TZVP basis set, and CTOCD-DZ method [85,86,101,102,103]. Time-dependent DFT calculations were performed with AMS (PBE0-dDsC/QZ4P) to explore the excited state properties. Spin-orbit coupling was taken into account by using the perturbational approach [104].

## 4. Conclusions

The halogen bond complexes CF${}_{3}$X⋯Y and C${}_{2}$F${}_{3}$X⋯Y, with Y = furan, thiophene, selenophene and X = Cl, Br, I, have been studied by using DFT and CCSD(T). It turns out that the PBE0-dDsC/QZ4P gives an adequate description of the interaction energies in these complexes, compared to CCSD(T) and experimental results. The energy decomposition analysis shows that all complexes are significantly stabilized by electrostatic, orbital, and dispersion interactions: not one factor dominates the interaction energy. In general, the interaction between the halogen atom and the π-bonds is stronger than with the lone pairs: the interaction is larger in the perpendicular orientation. The strength of the interaction follows the trend Cl < Br < I; the chalcogenide in the aromatic ring nor the hybridization plays a decisive role. Upon halogen bond formation, the aromaticity of the five-membered ring is unaffected: the π-ring current remains equally strong and diatropic in the complex as it is for the free aromatic ring. However, the photophysical properties of the complex are affected. The spin-orbit coupling between the singlet and triplet $\pi \to \pi^{*}$ states is increased, and a faster intersystem crossing is therefore expected. This effect of halogen bond formation can play a role in the formation of triplets in organic electronic devices when iodine containing additives are added.

## Figures and Tables

**Table 1:** Interaction energies (kJ/mol) for the parallel oriented complexes calculated with PBE0-dDsC/QZ4P and CCSD(T)-DLPNO/CBS.

		Furan		Thiophene		Selenophene	
		CCSD(T) CBS	DFT QZ4P	CCSD(T) CBS	DFT QZ4P	CCSD(T) CBS	DFT QZ4P
C${}_{2}$F${}_{3}$X	Cl	−7.2	−7.0	−9.1	−8.1	−0.5	−0.3
	Br	−9.4	−9.7	−8.5	−7.7	−10.8	−10.1
	I	−13.9	−12.1	−16.2	−12.7	−17.2	−13.8
CF${}_{3}$X	Cl	−7.0	−7.1	−5.1	−4.6	−9.6	−8.3
	Br	−9.1	−9.1	−8.0	−7.6	−8.7	−8.8
	I	−13.3	−11.5	−15.7	−12.4	−16.2	−13.9

**Table 2:** Interaction energies (kJ/mol) for the perpendicular oriented complexes calculated with PBE0-dDsC/QZ4P and CCSD(T)-DLPNO/CBS.

		Furan		Thiophene		Selenophene	
		CCSD(T) CBS	DFT QZ4P	CCSD(T) CBS	DFT QZ4P	CCSD(T) CBS	DFT QZ4P
C${}_{2}$F${}_{3}$X	Cl	−7.7	−7.2	−10.1	−8.9	−10.0	−8.7
	Br	−10.4	−10.1	−10.8	−9.9	−12.3	−11.5
	I	−15.0	−13.6	−17.7	−14.4	−17.8	−15.1
CF${}_{3}$X	Cl	−7.8	−7.7	−10.0	−9.0	−9.7	−8.8
	Br	−11.0	−10.9	−11.8	−10.9	−11.3	−10.9
	I	−14.5	−13.4	−16.6	−14.2	−17.5	−14.7

**Table 3:** Calculated (PBE0-dDsC/QZ4P) and experimental interaction enthalpies (kJ/mol) ${}^{a}$ for the parallel (‖) and perpendicular (⊥) oriented CF${}_{3}$I⋯furan complexes in the gas phase.

Compound	ΔH${}^{150K}$	ΔH${}^{298K}$	ΔH${}_{\exp}^{150K}$
CF${}_{3}$I⋯furan (‖)	−12.8	−14.0	−14.0(8)
CF${}_{3}$I⋯furan (⊥)	−12.0	−10.8	−14.4(9)

${}^{a}$ Gas phase complexation enthalpy obtained by correcting the experimental value in LKr for solvent effects. Corrections were introduced by using MC-FEP simulations similar to those described in [20].

**Table 4:** Energy decomposition analysis (EDA) in kJ/mol for the parallel oriented complexes.

**Complex**	** $E_{\mathrm{Pauli}}$ **	** $E_{\mathrm{els}}$ **	** $E_{\mathrm{steric}}$ **	** $E_{\mathrm{orb}}$ **	** $E_{\mathrm{disp}}$ **	** $E_{\mathrm{bond}}$ **
C${}_{2}$F${}_{3}$Cl⋯furan	7.0	−7.5	−0.5	−3.1	−3.0	−6.7
C${}_{2}$F${}_{3}$Br⋯furan	10.7	−11.2	−0.5	−5.1	−3.4	−9.0
C${}_{2}$F${}_{3}$I⋯furan	16.9	−16.7	0.2	−7.9	−4.2	−11.9
C${}_{2}$F${}_{3}$Cl⋯thiophene	7.0	−6.5	0.5	−3.5	−4.8	−7.8
C${}_{2}$F${}_{3}$Br⋯thiophene	10.2	−8.6	1.7	−5.4	−3.8	−7.5
C${}_{2}$F${}_{3}$I⋯thiophene	18.6	−14.0	4.6	−10.8	−6.1	−12.3
C${}_{2}$F${}_{3}$Cl⋯selenophene	−0.0	−0.2	−0.2	0.0	−0.3	−0.5
C${}_{2}$F${}_{3}$Br⋯selenophene	12.1	−10.1	2.0	−6.9	−4.8	−9.7
C${}_{2}$F${}_{3}$I⋯selenophene	23.6	−17.3	6.3	−13.3	−6.0	−13.1
CF${}_{3}$Cl⋯furan	7.5	−7.9	−0.4	−3.4	−2.9	−6.8
CF${}_{3}$Br⋯furan	9.6	−10.2	−0.7	−4.6	−3.2	−8.5
CF${}_{3}$I⋯furan	17.6	−16.9	0.6	−7.9	−4.1	−11.4
CF${}_{3}$Cl⋯thiophene	1.1	−2.5	−1.4	−1.2	−2.1	−4.7
CF${}_{3}$Br⋯thiophene	10.0	−8.3	1.8	−5.7	−3.2	−7.1
CF${}_{3}$I⋯thiophene	20.6	−15.1	5.6	−11.7	−5.8	−12.0
CF${}_{3}$Cl⋯selenophene	7.5	−6.6	0.9	−4.1	−4.8	−8.0
CF${}_{3}$Br⋯selenophene	14.5	−11.4	3.1	−7.8	−3.6	−8.2
CF${}_{3}$I⋯selenophene	28.5	−20.4	8.1	−15.9	−5.8	−13.6

**Table 5:** Energy decomposition analysis (EDA) in kJ/mol for the perpendicular oriented complexes.

**Complex**	** $E_{\mathrm{Pauli}}$ **	** $E_{\mathrm{els}}$ **	** $E_{\mathrm{steric}}$ **	** $E_{\mathrm{orb}}$ **	** $E_{\mathrm{disp}}$ **	** $E_{\mathrm{bond}}$ **
C${}_{2}$F${}_{3}$Cl⋯furan	5.6	−5.6	0.0	−2.9	−4.0	−7.0
C${}_{2}$F${}_{3}$Br⋯furan	13.1	−11.3	1.8	−6.4	−4.7	−9.4
C${}_{2}$F${}_{3}$I⋯furan	22.0	−17.8	4.2	−11.9	−5.8	−13.5
C${}_{2}$F${}_{3}$Cl⋯thiophene	8.2	−7.1	1.1	−4.0	−5.3	−8.3
C${}_{2}$F${}_{3}$Br⋯thiophene	8.7	−8.3	0.5	−4.8	−5.0	−9.3
C${}_{2}$F${}_{3}$I⋯thiophene	23.7	−18.3	5.4	−12.3	−7.1	−14.1
C${}_{2}$F${}_{3}$Cl⋯selenophene	7.7	−6.9	0.7	−3.8	−5.2	−8.3
C${}_{2}$F${}_{3}$Br⋯selenophene	14.0	−11.7	2.3	−7.3	−5.7	−10.6
C${}_{2}$F${}_{3}$I⋯selenophene	23.5	−18.6	4.9	−12.7	−7.0	−14.9
CF${}_{3}$Cl⋯furan	9.2	−8.0	1.2	−4.4	−4.1	−7.2
CF${}_{3}$Br⋯furan	16.4	−12.8	3.6	−8.4	−5.0	−9.8
CF${}_{3}$I⋯furan	24.0	−18.7	5.3	−13.1	−5.7	−13.4
CF${}_{3}$Cl⋯thiophene	8.2	−7.2	1.0	−4.2	−5.1	−8.3
CF${}_{3}$Br⋯thiophene	14.4	−11.6	2.8	−7.4	−5.4	−10.0
CF${}_{3}$I⋯thiophene	25.9	−19.9	6.1	−13.7	−6.7	−14.3
CF${}_{3}$Cl⋯selenophene	7.6	−7.1	0.5	−3.9	−5.0	−8.3
CF${}_{3}$Br⋯selenophene	16.2	−12.6	3.5	−8.2	−5.3	−9.9
CF${}_{3}$I⋯selenophene	27.6	−20.6	7.1	−14.3	−7.0	−14.2

**Table 6:** Excitation energies (eV) of the bright singlet and dark triplet $\pi \to \pi^{*}$ states and the spin-orbit coupling matrix element (cm${}^{-1}$) for CF${}_{3}$I⋯furan in parallel (‖) and perpendicular (⊥) orientation.

Compound	S($\pi \to \pi^{*}$)	T($\pi \to \pi^{*}$)	$\langle S|H_{\mathrm{SO}}|T\rangle$
furan	6.28	3.97/3.97/3.97	0.01
thiophene	5.85	3.74/3.74/3.74	0.06
selenophene	5.50	3.53/3.53/3.53	0.38
CF${}_{3}$Br⋯furan (‖)	6.29	3.97/3.97/3.97	1.05
CF${}_{3}$Br⋯furan (⊥)	6.35	4.00/4.00/4.00	14.32
C${}_{2}$F${}_{3}$Br⋯furan (⊥)	6.22	4.00/4.00/4.00	11.77
CF${}_{3}$I⋯furan (‖)	6.31	4.00/4.00/4.00	0.09
CF${}_{3}$I⋯furan (⊥)	6.29	4.03/4.03/4.04	23.73
CF${}_{3}$I⋯thiophene (⊥)	5.80	3.80/3.80/3.80	41.17
CF${}_{3}$I⋯selenophene (⊥)	5.44	3.61/3.61/3.61	68.56
C${}_{2}$F${}_{3}$I⋯furan (⊥)	6.24	4.03/4.03/4.04	6.84
C${}_{2}$F${}_{3}$I⋯thiophene (⊥)	5.85	3.80/3.80/3.80	64.62
C${}_{2}$F${}_{3}$I⋯selenophene (⊥)	5.49	3.61/3.61/3.61	67.56

## Data Availability

All inputs/outputs are available upon request.

## Supplementary Materials

The supporting information can be downloaded at: https://www.mdpi.com/article/10.3390/molecules28020772/s1

## Abbreviations

The following abbreviations are used in this manuscript:

CCSD(T)	Single double coupled cluster with perturbative triples
EDA	Energy Decomposition Analysis
CDFT	Conceptual Density Functional Theory
DFT	Density Functional Theory
DZ	Double Zeta
TZ2P	Triple-Zeta with two polarization functions
QZ4P	Valence Quadruple-Zeta + 4 polarization function, relativistically optimized
