# Supplementary material for: The Halogen Bond in Weakly Bonded Complexes and the Consequences for Aromaticity and Spin-Orbit Coupling
Source: Molecules. 2023 Jan 12;28(2):772. doi: 10.3390/molecules28020772 (PMC9865902; doi:10.3390/molecules28020772)
Supplement: Supplementary file 1 [file molecules-28-00772-s001.zip › molecules-2031474-supplementary.pdf]

1 Supplementary Information

1.1 Optimized geometries

In this part of the document the geometries (in Å) of all optimized complexes are shown.

Table S1: Geometry optimization with QZ4P basis of C2F3Br furan perpendic-ular

Atoms	x	y	z
C	3.015 064 93	1.165 218 32	0.050 606 11
O	3.594 174 32	0.674 819 43	−1.066 251 34
H	4.502 513 59	−1.106 649 82	−1.574 766 08
H	3.935 175 70	−1.821 549 56	1.001 359 30
H	2.680 339 56	0.376 813 28	2.047 488 74
H	2.627 866 40	2.166 243 43	−0.017 659 45
C	−2.847 512 35	0.408 073 85	0.106 552 95
C	−1.939 369 56	−0.482 932 53	−0.251 998 26
F	−2.572 424 47	1.555 984 83	0.666 787 56
F	−4.133 984 80	0.235 927 94	−0.060 951 08
F	−2.317 016 27	−1.622 042 84	−0.813 466 64
Br	−0.115 973 04	−0.247 210 76	−0.014 690 34

Table S2: Geometry optimization with QZ4P basis of C2F3Br furan parallel

**Atoms**	**x**	**y**	**z**
C	3.596 272 11	−1.014 976 19	0.033 078 33
O	2.659 664 24	−0.039 159 53	−0.001 128 82
H	2.703 387 20	2.024 031 46	−0.061 933 35
H	5.416 511 77	1.696 208 03	−0.037 839 77
H	5.766 415 03	−1.018 427 66	0.044 128 91
H	3.225 309 60	−2.023 994 20	0.060 824 80
C	−3.015 837 86	0.569 212 52	0.020 249 46
C	−2.190 523 28	−0.462 261 63	−0.020 161 34
F	−2.633 291 64	1.817 235 22	0.073 603 19
F	−4.319 290 80	0.451 407 42	0.010 253 59
F	−2.675 709 23	−1.694 351 32	−0.073 939 50
Br	−0.344 514 63	−0.312 430 29	−0.006 552 45

Table S3: Geometry optimization with QZ4P basis of C2F3Br selenophene par-allel

**Atoms**	**x**	**y**	**z**
C	3.164 725 21	−1.124 319 18	0.359 465 70
Se	2.847 814 71	−0.016 115 51	−1.088 466 91
H	2.729 819 58	2.415 421 97	−0.253 893 29
H	3.162 134 05	1.675 642 65	2.197 892 87
H	3.450 353 07	−0.888 734 26	2.466 325 35
H	3.247 787 07	−2.190 911 47	0.228 587 74
C	−3.300 633 39	0.568 873 10	0.169 081 08
C	−2.394 060 91	−0.375 939 12	0.348 361 85
F	−3.059 793 06	1.725 703 03	−0.388 112 78
F	−4.551 886 92	0.446 968 24	0.533 903 91
F	−2.737 725 03	−1.518 954 17	0.924 649 52
Br	−0.619 457 74	−0.219 865 02	−0.161 694 51

Table S4: Geometry optimization with QZ4P basis of C2F3Br selenophene per-pendicular

**Atoms**	**x**	**y**	**z**
C	−2.896 291 60	−0.789 585 22	1.117 496 85
Se	−2.961 539 64	−0.609 625 79	−0.720 684 20
H	−2.304 460 81	1.802 421 40	−1.333 779 76
H	−1.971 757 57	2.429 072 57	1.163 865 36
H	−2.429 765 38	0.459 932 71	2.791 720 93
H	−3.134 448 11	−1.730 755 92	1.585 569 68
C	3.310 777 97	−0.794 545 46	0.169 189 76
C	2.729 559 94	0.366 423 44	−0.078 023 01
F	2.673 123 97	−1.878 269 73	0.522 589 17
F	4.603 155 04	−0.986 330 87	0.085 586 40
F	3.467 290 53	1.409 903 23	−0.431 745 00
Br	0.903 468 99	0.659 920 98	0.033 941 78

Table S5: Geometry optimization with QZ4P basis of C2F3Br thiophene perpen-dicular

**Atoms**	**x**	**y**	**z**
C	−3.781 697 24	−0.288 233 97	−1.175 436 34
S	−3.361 544 97	1.198 545 73	−0.459 010 47
H	−2.395 668 94	1.018 868 93	1.767 207 14
H	−2.619 708 32	−1.595 025 47	1.660 812 47
H	−3.679 432 59	−2.367 861 57	−0.617 945 09
H	−4.216 243 43	−0.312 786 81	−2.161 262 92
C	3.148 466 15	0.452 908 79	0.223 593 66
C	2.252 795 47	−0.220 224 82	−0.477 660 67
F	2.854 944 08	1.311 755 33	1.163 291 43
F	4.440 135 02	0.329 852 84	0.052 327 39
F	2.648 812 01	−1.073 126 53	−1.410 889 70
Br	0.423 119 86	−0.061 015 80	−0.236 370 55

Table S6: Geometry optimization with QZ4P basis of C2F3Br thiophene parallel

**Atoms**	**x**	**y**	**z**
C	3.602 103 53	−1.051 778 53	−0.175 890 15
S	2.795 939 57	0.199 988 12	−1.006 259 27
H	2.961 018 05	2.389 694 70	0.042 058 89
H	4.419 128 54	1.362 856 07	1.970 227 36
H	4.781 961 02	−1.216 209 38	1.619 327 73
H	3.584 909 79	−2.057 545 88	−0.562 474 83
C	−3.194 561 96	0.456 776 06	−0.133 909 33
C	−2.308 829 13	−0.315 018 62	0.471 807 01
F	−2.889 696 69	1.414 547 66	−0.967 921 71
F	−4.487 048 10	0.342 254 84	0.036 623 67
F	−2.715 636 29	−1.264 866 45	1.300 386 34
Br	−0.477 796 53	−0.169 749 24	0.226 173 20

Table S7: Geometry optimization with QZ4P basis of C2F3Cl furan perpendic-ular

**Atoms**	**x**	**y**	**z**
C	3.338 247 50	1.283 677 20	0.073 572 48
O	3.682 476 72	0.587 109 27	−1.031 050 25
H	3.825 226 29	−1.415 392 84	−1.505 798 06
H	3.021 323 25	−1.808 681 72	1.076 373 36
H	2.713 609 69	0.722 867 60	2.076 494 09
H	3.367 570 77	2.354 956 72	−0.015 132 26
C	−2.713 063 24	0.427 309 16	−0.246 073 52
C	−1.966 501 96	−0.528 193 50	0.281 212 10
F	−2.237 795 21	1.528 337 08	−0.763 041 15
F	−4.018 538 25	0.369 231 40	−0.307 651 74
F	−2.535 563 74	−1.613 954 72	0.782 611 11
Cl	−0.279 366 19	−0.470 689 36	0.367 227 12

Table S8: Geometry optimization with QZ4P basis of C2F3Cl furan parallel

**Atoms**	**x**	**y**	**z**
C	−3.433 524 60	−1.043 244 01	−0.002 684 57
O	−2.507 946 81	−0.057 449 19	−0.006 304 20
H	−2.574 139 67	2.005 349 05	−0.006 812 93
H	−5.283 788 05	1.648 139 06	0.009 488 92
H	−5.604 014 32	−1.071 647 13	0.009 939 01
H	−3.051 420 67	−2.048 498 81	−0.005 646 22
C	3.016 379 77	0.504 418 76	0.002 145 18
C	2.182 379 22	−0.521 501 06	−0.000 811 45
F	2.639 252 45	1.755 323 60	0.006 712 82
F	4.318 115 57	0.375 491 17	0.000 810 45
F	2.654 669 31	−1.759 189 62	−0.001 369 63
Cl	0.499 451 50	−0.373 299 53	−0.003 960 60

Table S9: Geometry optimization with QZ4P basis of C2F3Cl selenophene par-allel

**Atoms**	**x**	**y**	**z**
C	−4.398 489 48	−0.859 650 90	0.788 373 47
Se	−2.823 100 69	−0.374 539 19	−0.047 194 51
H	−3.268 351 22	1.695 276 79	−1.506 026 16
H	−5.770 454 00	1.744 926 46	−0.808 678 31
H	−6.417 488 12	−0.149 581 51	0.841 512 42
H	−4.431 796 91	−1.709 446 99	1.450 601 86
C	3.829 734 38	0.984 021 39	0.040 404 34
C	4.322 315 17	−0.230 039 65	0.219 226 41
F	3.009 495 90	1.306 864 32	−0.922 730 99
F	4.115 620 09	1.996 928 45	0.815 683 63
F	5.150 595 92	−0.471 736 00	1.222 763 87
Cl	3.963 999 19	−1.548 127 87	−0.774 969 16

Table S10: Geometry optimization with QZ4P basis of C2F3Cl thiophene parallel

**Atoms**	**x**	**y**	**z**
C	3.456 508 97	−1.187 025 56	−0.422 846 10
S	3.155 374 72	0.303 965 59	−1.189 572 28
H	2.451 465 33	2.091 634 70	0.301 962 50
H	2.611 147 64	0.455 223 48	2.350 149 74
H	3.377 813 89	−1.943 205 19	1.594 383 15
H	3.773 372 62	−2.040 718 48	−0.999 096 30
C	−3.036 974 30	0.620 309 18	0.161 507 77
C	−2.131 238 63	−0.328 876 85	−0.003 050 64
F	−2.780 155 37	1.800 414 31	0.660 177 49
F	−4.296 868 55	0.477 999 68	−0.160 441 76
F	−2.483 193 08	−1.497 214 55	−0.518 569 12
Cl	−0.499 585 61	−0.161 095 84	0.402 507 34

Table S11: Geometry optimization with QZ4P basis of C2F3Cl thiophene per-pendicular

**Atoms**	**x**	**y**	**z**
C	2.948 526 72	1.183 436 99	0.083 144 31
S	3.108 250 38	0.055 357 62	−1.182 958 01
H	2.986 489 03	−2.274 132 62	−0.491 464 48
H	2.656 864 08	−1.524 808 94	2.002 451 54
H	2.654 892 83	1.096 284 66	2.216 575 03
H	2.986 029 84	2.240 673 16	−0.122 036 21
C	−2.767 518 70	0.721 116 54	0.068 774 96
C	−2.129 665 21	−0.427 970 98	−0.075 902 55
F	−2.192 274 68	1.839 339 92	0.422 853 88
F	−4.053 253 37	0.861 836 46	−0.128 129 30
F	−2.796 875 26	−1.514 106 55	−0.437 695 14
Cl	−0.471 588 62	−0.628 154 42	0.175 375 20

Table S12: Geometry optimization with QZ4P basis of C2F3I furan perpendicular

**Atoms**	**x**	**y**	**z**
C	−3.152 151 12	1.180 000 12	0.007 832 31
O	−3.765 391 79	0.667 472 10	1.094 765 61
H	−4.698 269 87	−1.119 949 50	1.534 742 40
H	−4.063 605 25	−1.777 878 91	−1.040 138 64
H	−2.778 437 47	0.441 725 66	−2.003 192 83
H	−2.766 953 05	2.179 270 69	0.107 671 45
C	3.026 954 18	0.406 538 09	0.198 423 74
C	2.105 791 54	−0.510 572 14	−0.046 180 24
F	2.778 499 65	1.680 231 04	0.354 479 66
F	4.304 101 24	0.141 714 49	0.312 555 98
F	2.482 522 33	−1.778 895 10	−0.186 515 40
I	0.091 154 85	−0.114 324 01	−0.231 654 23

Table S13: Geometry optimization with QZ4P basis of C2F3I furan parallel

**Atoms**	**x**	**y**	**z**
C	3.779 072 02	−1.006 775 10	0.019 385 79
O	2.809 211 26	−0.062 243 43	−0.008 488 78
H	2.781 875 86	2.002 642 84	−0.047 544 57
H	5.504 518 80	1.766 770 62	−0.014 917 38
H	5.947 046 48	−0.934 687 43	0.040 938 08
H	3.442 388 10	−2.027 954 10	0.034 777 77
C	−3.094 846 59	0.623 349 05	0.021 261 67
C	−2.301 571 76	−0.434 281 97	−0.020 046 10
F	−2.674 880 67	1.860 268 36	0.071 080 02
F	−4.401 998 76	0.552 074 62	0.016 893 50
F	−2.848 811 96	−1.646 385 12	−0.069 217 94
I	−0.246 362 75	−0.344 898 55	−0.013 506 34

Table S14: Geometry optimization with QZ4P basis of C2F3I selenophene parallel

**Atoms**	**x**	**y**	**z**
C	3.279 702 23	−1.147 676 93	0.672 251 17
Se	3.081 404 83	−0.348 450 90	−0.987 110 82
H	2.906 623 00	2.198 506 51	−0.657 779 65
H	3.144 823 81	1.959 003 45	1.917 285 33
H	3.400 709 72	−0.499 405 81	2.706 167 67
H	3.365 818 91	−2.218 415 30	0.760 014 67
C	−3.106 322 00	0.584 172 53	0.623 411 90
C	−2.483 818 37	−0.359 883 36	−0.062 521 53
F	−2.518 086 79	1.630 260 47	1.142 022 44
F	−4.393 869 20	0.581 139 10	0.862 636 20
F	−3.193 412 71	−1.378 727 38	−0.542 365 00
I	−0.459 560 53	−0.373 157 28	−0.449 298 81

Table S15: Geometry optimization with QZ4P basis of C2F3I selenophene per-pendicular

**Atoms**	**x**	**y**	**z**
C	3.077 630 57	0.854 885 43	1.125 711 59
Se	3.188 376 44	0.668 415 61	−0.709 279 00
H	2.548 437 51	−1.746 253 37	−1.328 820 66
H	2.152 380 01	−2.363 240 90	1.165 014 26
H	2.563 944 26	−0.385 100 53	2.793 442 19
H	3.302 553 91	1.798 051 28	1.596 399 53
C	−3.333 071 81	0.898 232 21	0.158 888 20
C	−2.771 710 54	−0.276 242 00	−0.075 563 56
F	−2.673 634 99	1.985 075 23	0.463 376 06
F	−4.624 705 24	1.109 210 10	0.112 362 30
F	−3.553 076 58	−1.311 378 47	−0.376 607 62
I	−0.744 687 86	−0.635 758 49	−0.002 287 45

Table S16: Geometry optimization with QZ4P basis of C2F3I thiophene parallel

**Atoms**	**x**	**y**	**z**
C	3.570 127 16	−1.114 577 52	0.514 958 51
S	3.415 995 64	−0.574 319 31	−1.095 937 39
H	3.374 398 28	1.845 907 94	−1.357 619 47
H	3.623 015 80	2.137 544 64	1.239 458 73
H	3.750 970 48	−0.191 045 22	2.452 008 48
H	3.593 508 86	−2.169 363 46	0.733 751 39
C	−2.618 498 26	0.415 984 71	0.811 583 26
C	−2.094 434 18	−0.170 152 32	−0.252 010 04
F	−1.923 083 56	0.996 418 18	1.753 959 78
F	−3.905 405 48	0.482 358 76	1.044 306 83
F	−2.908 333 11	−0.728 672 58	−1.144 385 15
I	−0.075 242 93	−0.289 145 32	−0.636 671 27

Table S17: Geometry optimization with QZ4P basis of C2F3I thiophene perpen-dicular

**Atoms**	**x**	**y**	**z**
C	3.378 201 23	1.273 980 96	0.545 419 60
S	3.325 781 33	0.743 453 44	−1.071 897 91
H	3.029 482 05	−1.656 525 76	−1.344 112 59
H	2.965 371 02	−1.953 085 15	1.264 609 12
H	3.235 087 89	0.357 627 14	2.491 835 11
H	3.504 858 57	2.320 244 40	0.770 358 93
C	−2.843 286 70	0.870 260 46	−0.076 931 09
C	−2.278 384 90	−0.306 630 63	0.136 114 14
F	−2.183 738 79	1.977 590 07	−0.295 827 40
F	−4.138 363 99	1.063 469 70	−0.093 504 66
F	−3.058 800 09	−1.364 259 43	0.347 513 81
I	−0.245 383 91	−0.629 731 38	0.165 939 36

Table S18: Geometry optimization with QZ4P basis of CF3Br furan perpendic-ular

**Atoms**	**x**	**y**	**z**
C	−2.770 897 71	−1.233 377 72	1.312 375 27
O	−2.322 954 50	−1.937 235 84	0.249 905 72
H	−2.251 960 51	−1.612 514 39	−1.786 817 68
H	−3.427 532 06	0.778 036 04	−1.174 329 87
H	−3.700 523 65	0.714 835 28	1.550 509 50
H	−2.657 496 58	−1.705 103 02	2.272 128 50
C	1.847 099 93	1.632 791 42	−0.460 865 66
F	1.791 819 09	2.667 011 42	0.361 186 77
F	2.259 557 86	2.058 468 04	−1.643 029 52
F	2.733 154 93	0.770 830 78	0.007 591 00
Br	0.116 218 54	0.804 448 62	−0.611 865 51

Table S19: Geometry optimization with QZ4P basis of CF3Br furan parallel

**Atoms**	**x**	**y**	**z**
C	−3.191 671 38	1.090 151 04	−0.073 290 80
O	−2.388 752 69	0.005 595 09	−0.173 140 73
H	−2.684 737 13	−2.037 103 20	−0.133 253 49
H	−5.316 354 87	−1.376 054 24	0.194 821 66
H	−5.327 932 88	1.362 697 24	0.193 926 03
H	−2.702 045 50	2.045 705 35	−0.134 752 96
C	2.581 627 85	−0.000 614 48	0.032 760 04
F	3.053 794 36	1.233 640 87	0.073 412 71
F	3.124 173 33	−0.624 240 15	−0.998 955 18
F	2.944 889 49	−0.625 829 95	1.139 556 85
Br	0.666 686 71	0.019 996 40	−0.125 556 82

Table S20: Geometry optimization with QZ4P basis of CF3Br selenophene par-allel

**Atoms**	**x**	**y**	**z**
C	2.838 867 43	0.255 860 61	1.258 716 52
H	2.723 992 74	0.212 478 23	−2.357 908 76
H	3.724 828 24	2.322 989 22	−1.223 804 07
H	3.734 947 70	2.195 126 48	1.367 772 16
H	2.741 261 26	−0.014 495 99	2.297 517 79
C	−3.082 225 66	0.203 124 82	−0.003 189 55
F	−3.423 847 85	0.622 075 08	1.203 056 59
F	−3.711 982 15	−0.933 321 47	−0.247 124 08
F	−3.477 401 92	1.101 454 01	−0.888 301 04
Se	2.279 119 36	−0.895 429 09	−0.077 496 31
Br	−1.175 334 38	−0.052 694 57	−0.107 180 43

Table S21: Geometry optimization with QZ4P basis of CF3Br selenophene per-pendicular

**Atoms**	**x**	**y**	**z**
C	1.961 454 40	0.247 091 33	−1.221 363 04
H	3.521 548 74	0.498 034 92	2.029 659 93
H	2.437 232 61	2.651 641 11	1.062 530 69
H	1.369 889 98	2.305 661 31	−1.277 450 85
H	1.600 559 71	−0.125 514 03	−2.166 247 52
C	−3.078 700 41	−0.122 131 63	0.116 686 84
F	−3.843 066 46	0.852 006 09	−0.348 075 39
F	−3.404 956 69	−1.243 232 89	−0.504 512 61
F	−3.325 622 79	−0.270 068 09	1.407 009 14
Se	2.862 586 82	−0.862 401 44	−0.049 778 23
Br	−1.220 984 47	0.286 036 87	−0.171 586 83

Table S22: Geometry optimization with QZ4P basis of CF3Br thiophene perpen-dicular

**Atoms**	**x**	**y**	**z**
C	2.559 640 76	−0.135 148 05	−1.244 373 11
S	3.155 501 84	−1.168 123 17	−0.027 967 21
H	3.728 122 48	−0.060 616 97	2.058 127 66
H	3.007 715 34	2.249 892 67	1.041 789 32
H	2.168 855 83	1.976 706 85	−1.438 110 81
H	2.280 547 92	−0.528 623 75	−2.208 032 76
C	−2.523 100 06	−0.057 184 83	0.167 682 50
F	−3.227 779 00	0.710 077 90	−0.646 940 88
F	−2.875 643 05	−1.315 710 35	−0.032 181 21
F	−2.821 120 68	0.269 546 73	1.413 748 23
Br	−0.641 803 50	0.172 926 26	−0.157 011 69

Table S23: Geometry optimization with QZ4P basis of CF3Br thiophene parallel

**Atoms**	**x**	**y**	**z**
C	3.229 767 25	0.019 557 20	1.242 783 50
H	2.952 847 96	−0.253 620 24	−2.243 462 05
H	4.443 688 37	1.716 225 53	−1.356 259 36
H	4.520 993 88	1.743 250 59	1.272 759 26
H	3.083 066 75	−0.204 961 31	2.286 546 70
C	−2.827 682 12	0.156 505 94	−0.017 713 63
F	−3.306 087 61	0.505 937 14	1.163 544 10
F	−3.414 182 37	−0.966 533 59	−0.394 068 04
F	−3.128 658 86	1.101 642 29	−0.891 051 72
S	2.491 645 16	−0.941 948 85	0.044 220 88
Br	−0.920 755 09	−0.085 448 03	0.075 820 78

Table S24: Geometry optimization with QZ4P basis of CF3Cl furan perpendic-ular

Atoms	x	y	z
C	−3.480 956 58	0.221 700 44	0.969 695 91
O	−3.233 678 17	−1.012 538 23	0.479 838 96
H	−2.448 050 61	−1.751 347 48	−1.278 091 35
H	−2.213 835 82	0.886 260 94	−1.965 007 21
H	−3.196 134 50	2.235 339 57	0.203 219 22
H	−3.912 563 37	0.260 149 03	1.954 032 89
C	2.337 773 12	0.006 303 50	0.071 386 98
F	2.759 677 00	1.253 674 05	−0.050 810 56
F	2.962 014 82	−0.737 037 25	−0.826 599 63
F	2.661 962 08	−0.430 207 05	1.276 258 66
Cl	0.605 826 82	−0.076 023 00	−0.164 839 73

Table S25: Geometry optimization with QZ4P basis of CF3Cl furan parallel

Atoms	x	y	z
C	−2.923 009 28	1.066 151 66	−0.036 610 46
O	−2.147 242 13	−0.039 507 35	−0.108 114 50
H	−2.503 294 82	−2.073 018 03	−0.100 248 31
H	−5.126 753 43	−1.339 817 96	0.110 994 64
H	−5.061 175 42	1.398 163 78	0.132 654 22
H	−2.405 541 60	2.008 394 96	−0.067 167 93
C	2.613 113 35	0.016 673 81	0.017 784 74
F	3.034 160 73	1.270 509 21	−0.001 179 66
F	3.140 641 41	−0.618 095 70	−1.015 552 96
F	3.049 684 24	−0.554 828 59	1.127 682 42
Cl	0.867 824 26	−0.041 545 51	−0.054 063 37

Table S26: Geometry optimization with QZ4P basis of CF3Cl selenophene par-allel

**Atoms**	**x**	**y**	**z**
C	−2.497 456 06	−0.019 777 29	1.209 963 89
H	−2.541 167 80	−0.148 538 20	−2.404 086 40
H	−2.491 467 08	−2.413 613 37	−1.132 215 92
H	−2.460 830 48	−2.146 013 40	1.448 079 21
H	−2.486 897 13	0.330 961 74	2.229 110 57
C	2.645 385 96	−0.373 057 24	0.029 822 65
F	3.317 268 79	0.373 533 32	0.890 483 36
F	3.225 671 61	−0.271 594 75	−1.154 444 30
F	2.717 376 23	−1.633 585 69	0.424 282 54
Se	−2.534 976 90	1.176 455 86	−0.199 325 34
Cl	0.978 719 14	0.144 646 94	−0.058 511 56

Table S27: Geometry optimization with QZ4P basis of CF3Cl selenophene per-pendicular

**Atoms**	**x**	**y**	**z**
C	−1.891 663 72	0.470 176 64	1.484 584 48
H	−2.669 579 84	0.115 433 88	−2.029 175 31
H	−1.793 566 10	2.426 055 84	−1.232 925 20
H	−1.284 858 69	2.515 634 83	1.311 053 21
H	−1.756 774 96	0.274 256 61	2.535 850 79
C	3.065 606 05	−0.054 067 14	−0.190 641 59
F	3.877 958 27	0.187 493 71	0.825 059 87
F	3.248 954 67	−1.302 824 61	−0.585 548 70
F	3.391 419 45	0.752 474 04	−1.187 482 89
Se	−2.523 017 81	−0.847 868 08	0.351 677 51
Cl	1.402 743 49	0.201 433 49	0.281 795 82

Table S28: Geometry optimization with QZ4P basis of CF3Cl thiophene parallel

**Atoms**	**x**	**y**	**z**
C	3.126 240 19	−0.950 928 16	0.779 497 13
H	2.925 971 00	1.470 506 97	−1.749 160 97
H	4.252 772 05	2.163 136 95	0.408 142 63
H	4.285 556 34	0.214 720 40	2.172 616 53
H	2.981 606 24	−1.887 572 52	1.292 355 70
C	−3.076 759 64	0.080 691 99	0.087 286 94
F	−3.427 072 80	−0.161 246 11	1.338 514 78
F	−3.727 260 12	−0.756 786 82	−0.701 873 52
F	−3.436 697 29	1.314 739 28	−0.220 224 89
S	2.479 284 41	−0.715 713 07	−0.779 223 06
Cl	−1.348 816 00	−0.116 172 35	−0.110 198 73

Table S29: Geometry optimization with QZ4P basis of CF3Cl thiophene perpen-dicular

**Atoms**	**x**	**y**	**z**
C	2.508 173 55	−0.378 195 91	−1.206 144 36
S	2.792 326 07	−1.126 069 72	0.297 109 75
H	2.926 482 48	0.401 229 82	2.185 242 02
H	2.513 887 66	2.436 194 56	0.578 928 93
H	2.229 261 97	1.640 437 39	−1.910 974 19
H	2.432 823 04	−0.970 513 06	−2.103 208 50
C	−2.482 122 29	0.023 823 75	0.222 487 26
F	−2.899 636 63	1.017 839 88	0.989 129 72
F	−3.256 404 62	−0.031 619 87	−0.848 677 47
F	−2.615 297 80	−1.107 382 84	0.894 402 13
Cl	−0.814 442 43	0.267 377 79	−0.239 534 62

Table S30: Geometry optimization with QZ4P basis of CF3I furan perpendicular

**Atoms**	**x**	**y**	**z**
C	3.917 830 85	−0.004 781 26	1.050 888 83
O	3.588 210 95	1.108 733 83	0.359 533 55
H	2.660 960 10	1.483 324 12	−1.444 307 64
H	2.488 106 40	−1.242 280 34	−1.610 893 44
H	3.656 729 29	−2.132 306 29	0.700 138 04
H	4.417 288 63	0.155 277 96	1.989 739 08
C	−2.481 480 79	0.007 271 84	0.142 975 89
F	−2.885 337 69	−1.075 913 33	0.791 240 71
F	−3.127 100 33	0.061 597 78	−1.013 421 15
F	−2.823 814 98	1.069 178 39	0.858 056 03
I	−0.357 115 72	−0.043 531 54	−0.173 697 92

Table S31: Geometry optimization with QZ4P basis of CF3I furan parallel

**Atoms**	**x**	**y**	**z**
C	−3.369 168 17	1.087 873 13	0.010 059 17
O	−2.567 516 66	−0.003 180 22	−0.005 975 51
H	−2.878 892 02	−2.044 670 65	−0.030 635 09
H	−5.526 249 96	−1.365 053 48	−0.011 436 15
H	−5.519 522 32	1.372 838 30	0.024 213 71
H	−2.868 847 25	2.039 789 61	0.019 383 82
C	2.644 383 55	0.002 297 77	0.001 435 79
F	3.108 671 80	1.242 692 61	−0.053 761 29
F	3.118 119 30	−0.663 081 14	−1.042 500 32
F	3.115 107 61	−0.566 672 90	1.102 143 88
I	0.502 288 78	−0.005 174 28	−0.001 633 49

Table S32: Geometry optimization with QZ4P basis of CF3I selenophene parallel

Atoms	x	y	z
C	2.968 306 12	0.130 839 83	1.223 348 17
H	2.933 456 76	0.250 025 16	−2.394 712 32
H	3.401 710 12	2.472 692 20	−1.131 663 91
H	3.387 062 09	2.215 450 53	1.449 123 17
H	2.908 527 12	−0.212 714 03	2.243 042 83
C	−2.786 409 99	0.349 416 37	0.000 805 34
F	−2.965 020 48	1.404 835 89	0.783 999 04
F	−3.528 758 32	−0.643 679 98	0.471 641 41
F	−3.228 994 57	0.656 027 39	−1.211 402 20
Se	2.711 768 25	−1.047 329 54	−0.183 375 76
I	−0.713 759 63	−0.210 491 60	−0.063 506 01

Table S33: Geometry optimization with QZ4P basis of CF3I selenophene per-pendicular

Atoms	x	y	z
C	2.273 088 45	0.352 495 53	−1.309 510 06
H	3.377 100 86	0.240 213 16	2.130 108 68
H	2.510 366 54	2.518 785 75	1.237 151 43
H	1.769 062 69	2.435 824 63	−1.248 451 77
H	2.036 632 84	0.087 648 01	−2.327 418 84
C	−2.984 112 04	−0.169 446 74	0.253 563 27
F	−3.831 759 04	0.659 895 63	−0.339 965 15
F	−3.290 149 16	−1.405 600 49	−0.116 294 84
F	−3.164 028 84	−0.074 757 32	1.563 926 51
Se	2.965 604 19	−0.898 721 26	−0.138 842 63
I	−0.958 554 22	0.302 287 05	−0.272 567 28

Table S34: Geometry optimization with QZ4P basis of CF3I thiophene parallel

**Atoms**	**x**	**y**	**z**
C	3.370 771 61	−0.032 348 76	1.276 424 18
H	3.110 165 13	−0.572 502 00	−2.180 883 32
H	3.755 576 86	1.860 206 73	−1.439 391 19
H	3.896 955 54	2.053 260 65	1.178 913 15
H	3.351 821 28	−0.239 109 10	2.333 764 58
C	−2.388 770 02	0.360 226 81	−0.009 904 68
F	−3.063 877 35	−0.122 305 61	1.023 763 95
F	−2.986 057 51	−0.049 236 60	−1.120 210 08
F	−2.457 587 54	1.683 460 08	0.032 807 31
S	3.019 525 01	−1.266 719 50	0.151 666 64
I	−0.347 944 90	−0.297 554 64	0.040 039 01

Table S35: Geometry optimization with QZ4P basis of CF3I thiophene parallel

**Atoms**	**x**	**y**	**z**
C	3.370 771 61	−0.032 348 76	1.276 424 18
H	3.110 165 13	−0.572 502 00	−2.180 883 32
H	3.755 576 86	1.860 206 73	−1.439 391 19
H	3.896 955 54	2.053 260 65	1.178 913 15
H	3.351 821 28	−0.239 109 10	2.333 764 58
C	−2.388 770 02	0.360 226 81	−0.009 904 68
F	−3.063 877 35	−0.122 305 61	1.023 763 95
F	−2.986 057 51	−0.049 236 60	−1.120 210 08
F	−2.457 587 54	1.683 460 08	0.032 807 31
S	3.019 525 01	−1.266 719 50	0.151 666 64
I	−0.347 944 90	−0.297 554 64	0.040 039 01

Table S36: Geometry optimization with QZ4P basis of CF3I thiophene perpen-dicular

**Atoms**	**x**	**y**	**z**
C	1.860 471 71	0.545 274 15	−1.896 921 80
S	3.279 289 51	−0.285 918 79	−1.451 719 42
H	4.612 636 32	0.821 854 15	0.255 272 61
H	2.882 255 46	2.789 970 44	0.346 916 64
H	0.905 098 88	2.399 338 81	−1.345 191 00
H	1.207 139 57	0.145 660 45	−2.655 244 40
C	−1.645 485 32	−1.366 402 41	1.891 922 36
F	−2.171 835 72	−0.476 737 34	2.722 835 42
F	−2.635 766 13	−1.944 906 66	1.226 185 61
F	−1.035 826 40	−2.295 696 86	2.614 900 11
I	−0.269 450 23	−0.423 610 50	0.542 580 33

1.2 Interaction energies with different basis sets.

Table S37: Interaction energies for the parallel oriented complex calculated with the PBE0 functional, and with DZP, TZ2P, QZ4P basis sets.

		Furan			Thiophene			Selenophene		
		DZP	TZ2P	QZ4P	DZP	TZ2P	QZ4P	DZP	TZ2P	QZ4P
**sp^2**	Cl	-14.6	-8.5	-7.2	-16.2	-8.3	-8.9	-16.2	-8.7	-8.7
	Br	-18.4	-11.4	-10.1	-19.9	-11.9	-9.9	-20	-13.2	-11.5
	I	-23.6	-15.2	-13.6	-24.5	-15.1	-14.4	-25.1	-16.4	-15.1
**sp^3**	Cl	-14.2	-8.7	-7.7	-15.7	-7.7	-9	-15.7	-8.5	-8.8
	Br	-17.7	-11.9	-10.9	-19.1	-11.9	-10.9	-19.1	-13	-10.9
	I	-23.1	-14.2	-13.4	-24.4	-14.8	-14.2	-24.6	-16.5	-14.7

Table S38: Interaction energies for the perpendicular oriented complex calculated with the PBE0 functional, and with DZP,TZ2P, QZ4P basis sets.

		Furan			Thiophene			Selenophene		
		DZP	TZ2P	QZ4P	DZP	TZ2P	QZ4P	DZP	TZ2P	QZ4P
**sp^2**	Cl	-14.6	-8.5	-7.2	-16.2	-8.3	-8.9	-16.2	-8.7	-8.7
	Br	-18.4	-11.4	-10.1	-19.9	-11.9	-9.9	-20	-13.2	-11.5
	I	-23.6	-15.2	-13.6	-24.5	-15.1	-14.4	-25.1	-16.4	-15.1
**sp^3**	Cl	-14.2	-8.7	-7.7	-15.7	-7.7	-9	-15.7	-8.5	-8.8
	Br	-17.7	-11.9	-10.9	-19.1	-11.9	-10.9	-19.1	-13	-10.9
	I	-23.1	-14.2	-13.4	-24.4	-14.8	-14.2	-24.6	-16.5	-14.7

Table S39: Interaction energies (kJ/mol) for the parallel oriented complexes cal-culated CCSD(T) DLPNO.

		Furan	Thiophene	Selenophene
	Cl	-7.2	-9.1	-0.5
sp^2	Br	-9.4	-8.5	-10.8
	I	-13.9	-16.2	-17.2
	Cl	-7.0	-5.1	-9.6
sp^3	Br	-9.1	-8.0	-8.7
	I	-13.3	-15.7	-16.2

Table S40: Interaction energies (kJ/mol) for the perpendicular oriented com-plexes calculated CCSD(T) DLPNO.

		Furan	Thiophene	Selenophene
	Cl	-7.7	-10.1	-10.0
Unsaturated (sp2)	Br	-10.4	-10.8	-12.3
	I	-15.0	-17.7	-17.8
	Cl	-7.8	-10.0	-9.7
Saturated (sp3)	Br	-11.0	-11.8	-11.3
	I	-14.5	-16.6	-17.5

Table S41: Harmonic frequencies of the $C_2F_3X \cdots$furan (∥) complexes.

$C_2F_3Cl \cdots$furan (∥)				
5.7	7.5	14.3	26.0	39.9
48.0	174.5	192.9	344.9	386.2
470.7	525.5	592.1	617.3	628.8
713.8	749.2	767.7	873.5	891.2
892.2	904.4	1023.3	1070.6	1087.8
1106.7	1176.1	1228.6	1239.0	1288.5
1358.9	1428.4	1531.9	1612.9	1865.2
3265.4	3275.8	3295.6	3302.8	
$C_2F_3Br \cdots$furan (∥)				
2.3	6.9	18.3	26.0	45.0
51.8	161.2	166.6	316.7	372.7
373.2	518.6	590.4	617.1	627.2
683.8	749.0	768.0	874.3	891.8
892.3	905.5	1023.8	1053.5	1070.2
1104.7	1176.1	1227.4	1228.6	1288.7
1353.8	1428.7	1532.8	1614.4	1853.7
3266.1	3276.6	3296.5	3304.2	
$C_2F_3I \cdots$furan (∥)				
10.9	12.8	20.9	37.0	51.3
53.5	148.7	152.3	292.6	325.9
362.8	515.5	593.9	616.3	625.5
669.4	748.1	768.0	875.2	891.3
892.7	906.9	1023.0	1034.7	1069.9
1101.8	1176.5	1209.1	1225.7	1289.0
1346.7	1428.7	1533.7	1616.4	1831.6
3266.8	3277.5	3297.2	3304.6	

Table S42: Harmonic frequencies of the $CF_3X\cdots$furan (‖) complexes.

$CF_3Cl\cdots$furan (‖)				
7.7	9.3	17.1	33.6	44.7
50.3	349.9	350.9	480.8	564.2
564.8	618.7	629.4	749.8	768.6
795.5	873.8	891.5	892.6	904.5
1023.8	1070.2	1102.5	1118.7	1176.3
1221.0	1221.8	1227.8	1288.6	1429.5
1532.8	1614.0	3266.4	3277.1	3296.7
3303.8				
$CF_3Br\cdots$furan (‖)				
5.1	11.8	27.8	40.5	46.2
49.3	304.8	305.1	354.6	551.5
551.7	618.4	628.1	748.8	768.5
771.0	874.8	891.8	892.1	906.0
1023.7	1070.1	1096.0	1108.7	1176.6
1217.4	1218.4	1227.2	1288.8	1429.5
1533.4	1615.0	3266.2	3276.8	3297.0
3304.0				
$CF_3I\cdots$furan (‖)				
3.4	16.1	16.9	34.7	50.7
59.9	269.8	270.3	290.4	540.5
541.0	615.6	625.6	749.2	754.9
768.2	874.5	890.9	893.1	906.3
1022.5	1070.7	1086.3	1104.7	1176.9
1194.1	1195.7	1226.4	1288.6	1428.9
1533.7	1615.7	3267.8	3280.0	3297.2
3305.1				

Table S43: Harmonic frequencies of the $C_2F_3X\cdots$furan ($\perp$) complexes.

$C_2F_3Cl\cdots$furan ($\perp$)				
1.9	3.8	10.4	21.9	28.2
51.4	174.1	188.7	343.3	387.1
470.1	524.9	593.7	620.8	631.7
714.1	753.0	771.2	873.9	888.9
893.2	904.5	1024.5	1070.7	1087.6
1109.5	1175.9	1229.2	1238.1	1288.1
1359.8	1430.1	1531.1	1610.4	1864.5
3266.0	3276.6	3296.6	3303.7	
$C_2F_3Br\cdots$furan ($\perp$)				
10.3	14.4	27.4	30.3	42.0
60.1	162.1	164.9	315.5	370.7
375.3	517.9	593.5	620.8	631.9
682.9	756.0	774.2	874.7	888.6
893.5	905.7	1024.4	1052.6	1070.8
1109.8	1175.5	1228.0	1229.5	1288.6
1353.5	1428.9	1529.7	1608.4	1853.1
3266.9	3277.3	3297.1	3304.0	
$C_2F_3I\cdots$furan ($\perp$)				
7.7	17.0	24.1	38.5	47.5
86.0	146.7	151.1	290.5	323.2
363.6	514.6	593.1	622.4	632.4
667.8	760.5	777.8	875.0	888.9
892.9	908.7	1023.3	1033.0	1070.9
1109.9	1175.0	1207.9	1228.9	1288.6
1345.3	1428.2	1528.2	1605.8	1829.5
3264.5	3275.7	3295.2	3301.3	

Table S44: Harmonic frequencies of the $CF_3X\cdots$furan (⊥) complexes.

$CF_3Cl\cdots$furan (⊥)				
2.7	13.0	25.7	35.0	39.8
59.5	349.2	349.6	477.2	564.2
564.5	622.0	631.3	755.2	774.0
794.0	874.8	888.8	893.0	907.2
1025.3	1070.9	1109.8	1112.8	1175.7
1221.1	1223.4	1230.6	1288.7	1429.1
1531.9	1609.3	3266.1	3277.1	3295.8
3303.6				
$CF_3Br\cdots$furan (⊥)				
8.6	22.1	34.4	43.6	45.0
69.3	303.4	303.9	349.7	550.9
551.3	620.4	631.8	757.0	769.1
773.7	876.9	888.9	893.2	906.2
1024.9	1070.4	1096.9	1110.5	1175.8
1215.3	1217.0	1229.9	1288.5	1429.3
1530.4	1607.4	3266.9	3277.6	3296.3
3307.7				
$CF_3I\cdots$furan (⊥)				
6.1	21.8	29.0	43.4	47.2
86.4	266.9	268.4	284.8	539.9
540.4	621.8	631.8	753.7	760.6
779.5	876.9	888.2	892.9	908.9
1025.1	1070.6	1088.8	1111.8	1175.1
1195.4	1197.6	1230.4	1288.7	1428.2
1528.0	1605.0	3266.7	3276.5	3296.8
3304.7				

Table S45: Harmonic frequencies of the $C_2F_3X\cdots$thiophene (‖) complexes.

$C_2F_3Cl\cdots$thiophene (‖)				
11.6	16.0	21.1	28.0	33.2
50.4	174.8	191.0	344.1	386.7
470.2	470.4	524.8	586.7	592.5
625.7	705.7	714.2	737.8	776.4
872.7	893.5	899.2	939.9	1069.9
1088.1	1097.9	1101.4	1237.8	1280.1
1358.7	1403.7	1459.9	1561.3	1865.3
3221.3	3234.6	3263.9	3267.3	
$C_2F_3Br\cdots$thiophene (‖)				
13.5	15.4	18.4	24.7	32.5
48.2	161.4	165.2	315.5	371.4
373.5	469.0	518.0	584.0	592.1
621.6	683.7	704.3	736.2	773.8
867.1	890.7	901.7	941.8	1053.1
1068.7	1098.5	1102.3	1228.7	1279.0
1355.4	1404.1	1461.7	1566.2	1853.4
3221.9	3235.3	3266.4	3269.7	
$C_2F_3I\cdots$thiophene (‖)				
15.8	16.1	19.6	24.4	40.0
62.0	146.0	149.8	291.6	324.4
360.2	468.8	514.8	582.8	592.5
623.6	668.5	705.3	737.6	772.3
865.2	891.7	904.5	944.0	1032.4
1068.3	1099.1	1103.0	1207.8	1280.1
1346.0	1403.8	1463.0	1568.7	1829.7
3224.6	3238.0	3267.1	3270.2	

Table S46: Harmonic frequencies of the $CF_3X\cdots$thiophene (‖) complexes.

$CF_3Cl\cdots$thiophene (‖)				
6.4	10.9	22.7	26.8	30.8
32.0	349.0	349.1	468.2	478.3
563.9	564.1	584.0	622.9	703.3
735.4	774.5	794.7	868.0	891.6
900.0	940.3	1068.1	1097.4	1101.5
1110.9	1222.8	1224.6	1278.4	1403.0
1459.7	1563.6	3221.7	3235.3	3265.5
3268.6				
$CF_3Br\cdots$thiophene (‖)				
14.3	17.0	28.4	36.8	39.0
56.8	302.9	303.7	351.2	471.9
551.0	551.3	583.8	623.5	704.2
735.4	770.0	773.9	867.5	892.4
902.4	942.0	1067.6	1095.3	1099.0
1102.4	1218.8	1219.4	1279.3	1404.0
1461.4	1566.7	3221.4	3235.0	3265.0
3267.8				
$CF_3I\cdots$thiophene (‖)				
11.5	12.7	23.6	31.3	38.1
52.5	267.6	268.1	287.6	466.6
539.7	540.3	582.6	622.8	705.5
737.5	754.0	771.6	864.4	890.9
904.8	944.1	1068.0	1087.0	1099.5
1103.4	1195.1	1195.4	1279.7	1403.7
1463.1	1568.8	3224.0	3237.2	3267.5
3270.3				

Table S47: Harmonic frequencies of the $C_2F_3X\cdots$thiophene (⊥) complexes.

$C_2F_3Cl\cdots$thiophene (⊥)				
10.9	14.8	16.1	35.6	43.1
51.8	174.1	189.7	343.7	386.5
470.7	471.2	525.0	585.3	591.8
625.6	705.6	714.7	737.7	775.9
870.2	892.8	899.5	939.5	1069.1
1088.4	1098.0	1102.0	1236.5	1280.2
1359.4	1404.1	1459.9	1562.7	1864.7
3221.8	3235.4	3265.0	3267.9	
$C_2F_3Br\cdots$thiophene (⊥)				
4.9	18.0	22.1	29.2	33.2
49.7	161.0	165.0	315.8	372.2
373.1	470.8	517.9	585.8	589.9
625.2	683.7	706.3	738.3	776.2
870.2	893.3	899.7	940.4	1053.0
1070.4	1098.1	1101.6	1227.7	1280.0
1354.3	1403.9	1459.9	1562.2	1853.2
3222.7	3236.2	3265.1	3267.9	
$C_2F_3I\cdots$thiophene (⊥)				
21.7	22.3	26.3	37.7	47.0
74.5	147.8	150.5	291.3	324.3
362.5	470.0	515.3	586.2	592.2
626.1	668.5	711.9	742.3	776.0
872.4	892.4	902.0	940.7	1032.9
1059.9	1097.0	1101.8	1206.9	1280.0
1345.2	1401.4	1455.4	1557.7	1829.3
3222.8	3232.8	3261.0	3265.7	

Table S48: Harmonic frequencies of the $CF_3X\cdots$thiophene ($\perp$) complexes.

$CF_3Cl\cdots$thiophene ($\perp$)				
15.9	16.6	24.2	39.2	45.1
51.4	348.9	349.5	471.0	479.9
564.1	564.5	587.1	625.8	705.9
738.0	776.2	794.3	871.1	893.4
899.4	939.9	1070.3	1097.6	1101.2
1113.0	1219.5	1220.8	1280.4	1403.9
1460.5	1562.1	3221.9	3235.6	3265.4
3268.3				
$CF_3Br\cdots$thiophene ($\perp$)				
3.3	20.2	24.0	34.3	37.2
61.4	303.0	304.2	351.9	471.2
550.9	551.1	586.5	625.4	708.0
741.0	770.6	776.2	870.5	893.5
900.9	941.3	1070.2	1096.5	1099.2
1100.9	1214.9	1215.6	1280.5	1402.8
1459.2	1558.5	3221.7	3235.1	3264.2
3267.4				
$CF_3I\cdots$thiophene ($\perp$)				
12.1	22.2	30.5	42.8	43.8
76.9	267.9	268.5	286.8	470.4
539.8	540.1	585.0	623.0	711.7
742.7	755.2	775.2	866.6	890.2
902.3	941.9	1068.9	1088.3	1097.3
1101.8	1193.8	1194.6	1279.8	1402.7
1456.4	1556.9	3222.6	3234.7	3264.9
3266.0				

Table S49: Harmonic frequencies of the $C_2F_3X\cdots$selenophene (∥) complexes.

$C_2F_3Cl\cdots$selenophene (∥)				
4.4	12.8	14.0	22.6	26.4
34.9	174.6	188.3	343.1	387.3
409.5	470.1	471.7	524.7	561.3
594.3	653.5	699.1	715.5	717.5
789.2	837.6	904.0	941.9	1047.8
1089.7	1095.8	1100.7	1240.3	1267.7
1362.9	1382.1	1480.8	1576.4	1865.9
3205.2	3220.4	3261.7	3264.3	
$C_2F_3Br\cdots$selenophene (∥)				
15.5	17.3	24.5	30.3	38.4
41.1	162.4	164.6	316.1	371.0
375.2	406.8	470.6	517.6	559.3
592.0	651.3	683.0	700.8	719.1
785.9	839.3	907.6	944.5	1046.6
1052.7	1096.1	1101.3	1225.7	1269.2
1353.1	1381.5	1484.3	1581.1	1853.5
3206.9	3220.9	3262.9	3265.2	
$C_2F_3I\cdots$selenophene (∥)				
14.6	21.6	27.4	34.8	36.8
44.8	146.4	150.9	290.8	322.8
362.6	406.7	469.3	514.7	559.5
592.2	649.5	667.9	701.9	720.2
784.5	839.5	910.6	946.9	1031.9
1046.5	1097.4	1102.0	1206.7	1269.8
1344.4	1381.3	1487.0	1584.4	1828.8
3208.6	3223.8	3264.0	3268.0	

Table S50: Harmonic frequencies of the $CF_3X\cdots$selenophene (‖) complexes.

$CF_3Cl\cdots$selenophene (‖)				
11.2	19.1	22.1	25.7	31.6
42.5	349.0	349.3	408.7	471.7
479.9	559.4	564.3	564.3	653.2
699.7	718.8	788.0	794.6	839.7
905.8	942.7	1047.3	1096.0	1100.8
1113.0	1219.0	1220.4	1269.8	1382.0
1482.8	1579.4	3206.9	3221.0	3261.2
3264.1				
$CF_3Br\cdots$selenophene (‖)				
9.4	14.1	17.3	30.1	36.2
42.5	302.7	303.7	348.5	409.0
469.2	550.8	551.3	559.4	650.1
699.5	717.9	769.4	786.0	838.1
908.0	944.4	1045.8	1094.9	1097.0
1101.4	1216.8	1218.4	1268.7	1381.5
1484.4	1581.3	3206.6	3220.2	3263.1
3265.7				
$CF_3I\cdots$selenophene (‖)				
12.0	18.1	26.0	31.8	34.3
46.9	267.3	267.5	284.1	405.8
469.6	539.0	539.6	557.0	648.7
701.2	719.7	752.3	783.8	839.4
910.7	946.7	1044.9	1086.1	1097.2
1101.9	1192.0	1192.8	1269.6	1380.4
1486.8	1584.5	3208.6	3221.9	3264.3
3266.6				

Table S51: Harmonic frequencies of the $C_2F_3X\cdots$selenophene (⊥) complexes.

$C_2F_3Cl\cdots$selenophene (⊥)				
11.5	14.5	26.5	31.7	33.2
50.1	175.5	189.6	344.0	389.2
410.5	471.2	471.9	525.2	562.3
594.3	653.9	701.5	714.9	720.8
789.3	839.8	905.0	942.3	1048.7
1088.8	1096.5	1101.6	1236.8	1269.7
1360.1	1382.5	1480.3	1575.7	1865.4
3207.4	3221.3	3262.4	3265.3	
$C_2F_3Br\cdots$selenophene (⊥)				
11.5	17.5	18.1	31.5	35.2
64.8	161.7	161.8	316.4	370.9
372.8	412.2	471.5	518.3	563.8
590.7	653.1	683.0	704.4	723.7
789.1	839.4	906.0	942.8	1047.8
1052.8	1096.5	1100.7	1225.6	1269.7
1354.1	1382.4	1480.4	1575.3	1851.4
3207.9	3222.2	3261.4	3265.2	
$C_2F_3I\cdots$selenophene (⊥)				
12.6	22.5	27.2	38.0	42.9
75.7	146.9	150.6	292.1	323.4
362.8	412.9	472.1	515.0	564.5
593.0	653.2	668.2	706.8	725.5
789.3	839.6	906.9	943.9	1033.1
1047.2	1096.7	1100.8	1205.4	1270.0
1345.6	1382.1	1478.4	1572.7	1828.4
3208.1	3222.7	3260.8	3265.0	

Table S52: Harmonic frequencies of the $CF_3X\cdots$selenophene ($\perp$) complexes.

$CF_3Cl\cdots$selenophene ($\perp$)				
13.8	14.2	21.5	29.6	36.6
49.0	348.8	349.7	410.3	470.7
479.9	561.4	563.9	564.3	652.3
701.0	720.2	788.5	794.8	838.4
905.0	942.2	1046.9	1096.2	1100.8
1112.7	1219.1	1220.3	1269.0	1382.0
1480.3	1576.2	3206.9	3220.6	3261.7
3264.8				
$CF_3Br\cdots$selenophene ($\perp$)				
7.5	13.7	16.9	28.5	34.1
71.5	303.3	303.8	350.8	413.2
470.2	550.9	551.4	563.4	652.6
704.8	723.5	769.5	789.5	838.4
905.7	942.6	1047.0	1096.2	1098.0
1101.4	1215.0	1217.0	1269.0	1382.0
1478.8	1574.3	3206.1	3220.8	3261.4
3264.8				
$CF_3I\cdots$selenophene ($\perp$)				
5.2	12.5	27.9	31.7	46.1
75.7	267.4	268.5	286.7	409.3
468.1	538.9	540.5	564.1	649.7
707.7	727.3	752.9	789.3	836.8
907.1	944.1	1047.7	1087.1	1097.1
1101.5	1192.4	1194.3	1268.0	1382.1
1477.1	1570.3	3206.9	3221.4	3261.1
3264.0				
